# Do Major Pharmacovigilance Databases Support Evidence of Second Trimester NSAID and Third Trimester Paracetamol Fetotoxicity?

**DOI:** 10.3390/ph17121592

**Published:** 2024-11-26

**Authors:** Katarina Dathe, Carolin Benndorf, Simone Bergner, Christof Schaefer

**Affiliations:** 1 Charité—Universitätsmedizin Berlin, Corporate Member of Freie Universität Berlin and Humboldt-Universität zu Berlin, Institute of Clinical Pharmacology and Toxicology, Embryotox Center of Clinical Teratology and Drug Safety in Pregnancy, Augustenburger Platz 1, 13353 Berlin, Germany; carolin.benndorf@charite.de (C.B.); christof.schaefer@charite.de (C.S.); 2 Federal Institute for Drugs and Medical Devices, Pharmacovigilance Division, Kurt-Georg-Kiesinger-Allee 3, 53175 Bonn, Germany; simone.bergner@bfarm.de

**Keywords:** anti-inflammatory agents, non-steroidal [MeSH], acetaminophen [MeSH] pregnancy trimester, second [MeSH], pregnancy trimester, third [MeSH], ductus arteriosus [MeSH], stillbirth [MeSH], oligohydramnios [MeSH], renal insufficiency [MeSH], persistent fetal circulation syndrome [MeSH], drug-related side effects and adverse reactions [MeSH]

## Abstract

**Background:** Paracetamol and non-steroidal anti-inflammatory drugs (NSAIDs) are frequently used during pregnancy. Due to their fetotoxicity, NSAIDs are contraindicated during the third trimester. There is ongoing controversy about the extent to which NSAIDs may cause cardiovascular and renal impairment in the fetus earlier in the second trimester. Paracetamol, used as an effective treatment for closure of patent ductus arteriosus (PDA) after birth, is suspected to cause similar but unwanted effects during the third trimester of pregnancy. **Methods:** Three major pharmacovigilance databases (VigilanceCentral, EudraVigilance, and VigiBase) were searched for Individual Case Safety Reports (ICSRs; *n* = 1288) on fetotoxic effects that have been shown to result from NSAID exposure in late pregnancy. **Results:** In 219/1288 cases, an NSAID and/or paracetamol was taken after the first trimester, and the ICSR was not related to other reported risk factors. Out of these 219 ICSRs, 48 were exposed to NSAIDs in the second but not the third trimester or to paracetamol in the third trimester. Causality assessment was “probable or likely” in four NSAID reports and none of the paracetamol reports. **Conclusions:** The scarcity of adverse drug reactions (ADRs) in our study and in the literature, despite decades of pharmaceutical marketing and worldwide use of paracetamol as an analgesic of choice in the third trimester and the absence of formal contraindications against NSAIDs in the second trimester, speaks against a substantial cardiovascular and nephrotoxic risk of temporary use of NSAIDs in the second trimester or paracetamol in the third trimester. NSAIDs continue to be contraindicated in the third trimester.

## 1. Introduction

Non-steroidal anti-inflammatory drugs (NSAIDs) combine analgesic, antipyretic, and anti-inflammatory properties. They are frequently used drugs, even during pregnancy. According to international guidelines for medication use in pregnancy, ibuprofen, as the most experienced NSAID in pregnancy, is one of the analgesic and anti-inflammatory drugs of choice during the first half of pregnancy. In single doses, it is also acceptable from gestational weeks (GW) 20 to 28. Paracetamol is considered the analgesic of choice throughout pregnancy (e.g., [1,2,3,4,5]). However, controversy regarding paracetamol and long-term neurodevelopmental effects, such as attention-deficit hyperactivity disorder (ADHD) and autism spectrum disorder (ASD), has led to increased caution in its use during the last decade [6,7,8,9]. More recent studies have reported that the observed associations of maternal paracetamol use with offsprings’ neurodevelopmental outcomes were nullified in the sibling comparison design [10,11].

Cardiovascular and renal fetotoxicity is evidenced for third trimester NSAID use. In particular, narrowing of the ductus arteriosus Botalli, associated cardiac complications, and renal impairment resulting in reduced amniotic fluid may occur after NSAID exposure in the third trimester of pregnancy [12,13,14,15,16]. It is, however, still a matter of debate whether NSAID use should be discouraged during the second trimester, and, if so, from which gestational week [17]. Furthermore, paracetamol is suspected to cause similar cardiovascular toxicity during the third trimester of pregnancy, as it is therapeutically effective in closing persistent ductus arteriosus (PDA) in newborn infants [18,19]. To further explore these risks, we decided to search the major pharmacovigilance databases for case reports supporting the hypotheses of fetal cardiovascular and/or renal toxicity of NSAID use during the second trimester and of paracetamol use during the third trimester of pregnancy. Paracetamol has been recommended for decades as an analgesic of first choice throughout pregnancy, especially during the third trimester, and NSAIDs, particularly ibuprofen, are established as anti-inflammatory and analgesic medication in the second trimester. Users and prescribers would therefore feel highly motivated to report adverse drug reactions (ADRs) that would question this treatment practice.

## 2. Results

**Figure 1:**
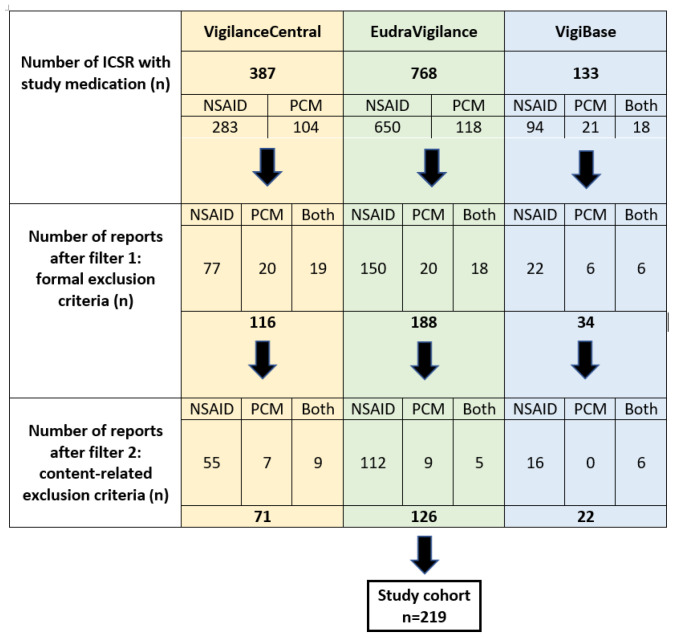
Flow chart of the selection process for generating the final study cohort. Filter 1 and filter 2 criteria are listed in detail in Table S1. ICSR, Individual Case Safety Report; *n*, number of cases; Both, co-exposed to NSAID/PCM; NSAID, non-steroidal anti-inflammatory drug; PCM, paracetamol.

Overall, *n* = 1288 Individual Case Safety Reports (ICSRs) from three pharmacovigilance databases were checked in detail (Figure 1). Pre-defined selection criteria were applied in a two-step process. In the first step (filter 1), all 1288 were checked for formal criteria, e.g., verification of exposure to study medication after the first trimester, exclusion of duplicates. In the second step (filter 2), cases with potential confounders relevant to our study endpoints were excluded, e.g., multiple malformations and/or genetic diseases, severe maternal illness, or infectious diseases. Criteria applied to generate the study cohort can be found in Table S1. The study cohort resulted in *n* = 219 pregnancies, of which *n* = 25 were twin pregnancies.

### 2.1. Exposure to Study Medication in the Second/Third Trimester

Cases retrieved from the three databases were assigned to three groups: NSAID exposed only, paracetamol exposed only, and NSAID plus paracetamol exposed. In 41/219 (18.7%), exposure to the study medication took place in the second trimester only; in 37 (16.9%), exposure occurred in the second and third trimesters; and in 126/219 (57.5%), exposure occurred in the third trimester only. In total, 15/219 (6.6%) were not clearly assignable to the second or third trimester (Table S2). The duration of use varied greatly from daily use (about 13%) to long-term use of at least three weeks (about 17%). The exposure pattern was either continuous or intermittent. Information on duration was missing or imprecise in about a quarter of the cases. For both, normal doses of NSAIDs and paracetamol were used. Cases with overdose or low-dose treatment with ASA for anticoagulation were excluded from the study cohort.

Table 1 gives an overview of the study medication by substance for cases exposed to NSAIDs. The most frequently reported NSAIDs were diclofenac (76/217; 35.0%), indomethacin (57/217; 26.3%), nimesulide (23/217; 10.6%), and ibuprofen (22/217; 10.1%).

### 2.2. Treatment Indication for Study Medication

Acute pain was the most common indication (37.4%), followed by chronic and/or inflammatory pain and threatened preterm labour (both 16.4%) (Table S3).

### 2.3. Study Endpoints

A brief summary of all reported pre- and postnatal study endpoints is provided in Table 2. Multiple endpoints in one case were counted and listed separately, and different outcomes were possible in twins, as they may react individually to external factors, e.g., due to different genomes. Case numbers in the following Section 2.3.1 and Section 2.3.2 refer to Table S4, with further details and causality assessments.

#### 2.3.1. Prenatal Study Endpoints

Ductus arteriosus stenosis was reported in 99/219 pregnancies. Of these, 11 pregnancies were exposed in the second trimester to NSAIDs, including 1 with NSAIDs plus paracetamol (Table S4: cases 1–11). Seven pregnancies were reported after paracetamol use in the third trimester (cases 38–44). After a thorough review of the given information, causality was concluded as “probable/likely” in one study case after diclofenac use for 6 days in GW 26/27 (case 1). Causality was assessed as possible in further reports (Table S4).

Cardiac impairment was present in 73/219 pregnancies. Five cases with this diagnosis occurred after second trimester NSAID exposure. In four of them, ductus arteriosus stenosis had also been diagnosed, which may have resulted in cardiac dysfunction (cases 1, 5, 8, 10). Another pregnancy complicated by severe maternal condition and oligohydramnios ended in a stillbirth (case 12). Cardiac impairment was also reported in four pregnancies with third trimester paracetamol use, all in combination with ductus arteriosus stenosis (cases 38, 40, 43, 44).

Fetal death occurred in 40/219 pregnancies. Among them, seven pregnancies were exposed to NSAIDs in the second trimester, of which one included NSAID plus paracetamol exposure (cases 5, 12–17). In none of these cases was a causal relationship between medication and diagnosis assessed as “probable/likely” or “certain”.

Oligo-/anhydramnios was reported in 76/219 pregnancies, including 21 pregnancies with NSAID exposure in the second trimester (cases 6, 7, 12–14, 16, 18–30, 34, 35) and 3 with NSAID plus paracetamol exposure (cases 31, 32, 33). Two pregnancies were reported after paracetamol use in the third trimester (case 43, 45). Causality was assessed as “probable/likely” in three second trimester NSAID-exposed cases: long-term use of indomethacin with intermittent ibuprofen for ankylosing spondylitis (case 18), nimesulide for more than three weeks for tocolysis (case 19), and naproxen for 5 days at GW 25 with improvement of oligohydramnios shortly after naproxen discontinuation (case 20).

#### 2.3.2. Postnatal Study Endpoints

Neonatal cardiac failure was reported in 66 infants out of 219 pregnancies. Of these, an exposure to second trimester NSAID use was reported in three pregnancies (cases 10, 36, 37), and third trimester paracetamol exposure was reported in five pregnancies (cases 38, 39, 41, 44, 46). In the majority of these infants (46/66, 69.7%), ductus arteriosus stenosis or cardiac abnormalities were already seen prenatally.

Persistent pulmonary hypertension (PPHT) was reported in 50 infants. Of these, two had second trimester NSAID exposure (cases 36, 37), and three were reported with third trimester paracetamol exposure (cases 38, 39, 46). PPHT was associated with prenatal ductus arteriosus stenosis or other cardiac dysfunction before or after birth in 39/50 infants.

Renal failure was diagnosed in 26 infants. Of these, second trimester NSAID exposure was reported in two cases, and both had a diagnosis of oligohydramnios in utero (cases 13, 30). One study case was reported with paracetamol exposure in the second/third trimester. The pregnancy was complicated by polyhydramnios requiring treatment (case 48).

In total, 19 infants in the study cohort died during the neonatal period. An exposure to NSAIDs in the second trimester was reported in 6/19 pregnancies (cases 2, 5, 13, 30, 35, 36), and five of these six infants were twins.

## 3. Discussion

Out of 219 pharmacovigilance cases with prenatal NSAID and/or paracetamol exposure and with one or more of predefined fetotoxic effects, 48 study cases correspond to our study focus on second trimester but not third trimester NSAID exposure or third trimester paracetamol exposure.

NSAIDs are known for their third trimester fetotoxic risks of ductus arteriosus stenosis [12,13] and renal impairment leading to decreased amniotic fluid [20,21]. Intrauterine narrowing or obstruction of the ductus arteriosus can lead to right heart failure, hydrops fetalis, and intrauterine death [22,23]. Less frequently, similar adverse effects may occur after NSAID exposure in the late second trimester. Several case reports of fetuses presenting with intrauterine ductal constriction after second trimester NSAID exposure were identified by a literature review [17]. From this knowledge, restrictions have been placed on the use of NSAIDs after 20 weeks’ gestation, with the exception of the use of low-dose aspirin [24,25].

Paracetamol is an often-used analgesic and antipyretic during pregnancy. Due to the fact that paracetamol, albeit for this indication at a much higher dose, seems to be similarly effective in the treatment of PDA in preterm infants as indomethacin or ibuprofen [18,19], there is concern that paracetamol may adversely close the ductus arteriosus during the third trimester of pregnancy. While case reports by Becqet et al. and a case series analysis by Allegaert et al. have sparked this discussion [26,27], a cohort study consisting of 604 prospectively evaluated paracetamol-exposed pregnancies in the third trimester showed that the risk of ductus arteriosus stenosis appears to be very low or even negligible [28]. An expert review on the topic came to the same conclusion after a critical literature review [29]. Hauben et al. comprehensively reviewed and evaluated experimental and clinical data, including reports from the US FDA Adverse Event Reporting System (FAERS) database. They concluded that paracetamol may at least sometimes contribute to ductus arteriosus narrowing [30]. Allegaert et al. proposed a paracetamol-induced decrease of placental prostaglandin production rather than a direct paracetamol effect on the fetus [31].

There was only one case of ductus arteriosus constriction with second trimester NSAID exposure in our study (Table S4, case 1) for which causality was assessed as “probable/likely” according to the WHO-UMC classification. Diclofenac was used for 6 days in GW 26 and 27 and ductus arteriosus constriction, dilatation of the right ventricle, and mild insufficiency of the tricuspid valve normalized after diclofenac discontinuation. Among third trimester paracetamol-exposed cases with ductus arteriosus constriction, two were assessed as possibly related to drug exposure. In one case, a high dose of 4000 mg/d paracetamol was taken from GW 30 for 14 days (Table S4, case 38), and in a second case, paracetamol was used at an unknown dose for 28 days from GW 36 (case 39).

In order not to miss an unrecognized ductus arteriosus closure in fatal outcomes, we included fetal death as an endpoint. However, there were no reports assessed as “probable/likely” or “certainly” related to the study medication.

Prenatal constriction of the ductus arteriosus Botalli may result from NSAIDs’ inhibition of prostaglandin synthesis. Ductus arteriosus constriction was observed even after topical use of NSAID [32]. Prenatal stenosis of the ductus arteriosus can also occur spontaneously. However, reliable data on prevalence are hardly available to date. A case series by Leal et al. described five fetuses with prenatally diagnosed occlusion of the ductus arteriosus, three of which occurred without prior maternal medication [33]. Luchese et al. found 20 fetuses with prenatal ductal stenosis in a cohort of 7000 pregnancies, of which 13/7000 (0.2%) were assessed as idiopathic [34]. Enzensberger et al. presented three other cases of prenatal idiopathic ductal stenosis in which the mothers had not taken prostaglandin inhibitors [35]. In another echocardiography study, 45 fetuses in 26,000 pregnancies were reported with ductal stenosis or obstruction. Of these, 8/26,000 cases were categorised as “spontaneous”, without any prior maternal medication [14].

Reduced amniotic fluid is assumed to result from NSAID-induced inhibition of prostaglandin synthesis in the fetal kidney, which lowers renal blood flow and tubular function, resulting in a reduction in fetal urine production [36]. NSAIDs may also influence kidney development and lead to irreversible structural alterations. In general, oligohydramnios occurs in 3–5% of pregnancies and can have multiple causes, such as placental insufficiency, premature rupture of the membranes, renal or urinary tract malformations, maternal treatment with ACE inhibitors or angiotensin II type 1 receptor blockers, fetal chromosomal anomalies, or intrauterine infections [37,38]. For the majority of study cases diagnosed with oligohydramnios, the relationship to second trimester NSAID exposure was assessed as possible, whereas in three cases causality appeared to be “probable/likely” (Table S4, case 18, 19, 20).

With respect to the other predefined postnatal fetotoxic endpoints, such as PPTH, PDA, other neonatal cardiac dysfunction, or renal failure, for second trimester NSAID and third trimester paracetamol exposure, no cases were assessed as “probable/likely” or even “certainly” related to the study medication.

The analysis performed for this study based on major pharmacovigilance databases has both strengths and limitations. Data from spontaneous reporting systems are a valuable source for identifying unusual and unexpected ADRs. EudraVigilance and VigiBase contain data from different populations and a large number of reporters from many nations. These ICSR data are frequently imprecise and incomplete. Evaluation of such datasets requires a case-by-case analysis with expertise in obstetrics, pediatrics, pharmacology, and human genetics. As ICSRs are spontaneously reported, they often lack demographic and comprehensive clinical information on patients, making it difficult or impossible to establish a causal relationship between drugs and the observed adverse effects. In addition, ADRs might have been misclassified by the reporter. We cannot rule out that our analysis is biased by unreported or missing case information or even unrecognized cases relevant to be reported as ICSRs. Therefore, our results should be carefully interpreted in light of other studies with different datasets and methodologies.

Pharmacovigilance databases lack comparison cohorts exposed to other drugs used for the same treatment indication to detect increased risks of the study drug. Furthermore, no denominator, i.e., an overall cohort of pregnant women with second trimester NSAID or third trimester paracetamol exposed pregnancies independent of pregnancy outcome, is available. Research exclusively based on spontaneous reporting to pharmacovigilance databases does not allow for quantification of risks.

Population studies based on prescription data suffer from the fact that NSAIDs and paracetamol, as over the counter (OTC) drugs, are only incompletely covered, a limitation that is less relevant for ADR-based studies. Finally, we are aware that the limited sample size of our study cohort (*n* = 219) makes it difficult to generalize the results. Structured and well-planned studies with appropriate comparison cohorts, information on potential confounders, and advanced computational statistics are needed to further clarify the presumably low fetotoxic risk of temporary use of NSAIDs in the second trimester and of paracetamol use in the third trimester of pregnancy.

Considering our research question, high numbers of ADRs would have supported a signal of relevant fetotoxicity. However, plausible ADRs with severe or even fatal outcome classified as “probable/likely” related to the drugs under study remain scarce in our study and in the literature, despite decades of pharmaceutical marketing and relatively unrestricted use of paracetamol throughout pregnancy and of NSAID use in the second trimester. This speaks against a substantial cardiovascular and nephrotoxic risk of NSAIDs in the second trimester and of paracetamol in the third trimester, at least when used temporarily and at therapeutic dosages. Nevertheless, NSAIDs in the second trimester after 20 weeks’ gestation and paracetamol in the third trimester should only be used if clearly indicated and preferably in single doses or for a few days only. NSAIDs should not be used in the third trimester.

## 4. Materials and Methods

### 4.1. Data Retrieval

For this study project, a search query was performed for spontaneous ADR reports in three pharmacovigilance databases: 1. VigilanceCentral, the national ADR database of the German Federal Institute for Drugs and Medical Devices (BfArM), including national reports until 22 November 2017; 2. EudraVigilance, the European ADR database of the European Medicines Agency (EMA); and 3. VigiBase, the ADR database maintained by the World Health Organization—Uppsala Monitoring Centre (WHO-UMC).

### 4.2. Search Strategy Within Databases

To identify ADR reports relevant to the research question, the search strategy was focused on the study medication using the according ATC-codes: NSAID (grouped under ATC-code M01A), acetylsalicylic acid (ASA, ATC-code N02BA01), and paracetamol/acetaminophen (ATC-code N02BE71). All study cases had to be exposed to at least NSAIDs, ASA, and/or paracetamol. Only cases with ASA exposure > 300 mg/day were considered.

This study focused on fetal effects after exposure to the study medication in the second and/or third trimester. ADR reports with one or more of the pathological findings listed below and classified as “suspected” in relation to the study medication were searched for. Included were the following:

Prenatal study endpoints

- Ductus arteriosus Botalli stenosis/closure.
- Fetal cardiac dysfunction/impairment.
- Oligohydamnios/anhydramnios.
- Stillbirth/fetal death in the second or third trimester.

Postnatal study endpoints

- Neonatal cardiac failure.
- Primary pulmonary hypertension (PPHT).
- Patent ductus arteriosus Botalli (PDA).
- Renal dysfunction.
- Neonatal death.

Standardized Medical Dictionary for Regulatory Activities (MedDRA) Queries were used. A total of 22 MedDRA Preferred Term (PT) codes in the Lowest Level Terms (LLTs) category were identified for the database query (Table S5).

VigilanceCentral and EudraVigilance was searched in February 2018 and March 2018, respectively; VigiBase was screened in February 2018. Data exports were generated, and the Individual Case Safety Reports (ICSRs), including the case narratives, were downloaded as separate PDF files. If available and referenced within ICSRs, corresponding publications were also considered.

### 4.3. Data Review and Filtering

All available datasets and documents were reviewed in detail. A two-stage process was performed to generate the relevant study cohort for further analyses and assessment (Table S1). Reports may have been registered more than once. Therefore, during the thorough data check of all datasets and relevant information, duplicate reports were excluded, and the available information was merged. Reports were considered relevant only if exposure occurred before the outcome was diagnosed, not after.

### 4.4. Study Cohort Analysis

Detailed information was carefully extracted from each ADR report. Analyses focused on details of drug exposure, the indication for drug use, and the various pre- and postnatal study endpoints. Those pregnancies with a relevant prenatal study endpoint and definitely exposed in the second trimester to NSAIDs or to paracetamol in the third trimester were re-evaluated case by case using the World Health Organization Uppsala Monitoring Centre (WHO-UMC) system for standardized case causality assessment [39]. The WHO-UMC categories of causal relationship are certain, probable/likely, possible, unlikely, conditional/unclassified, and unassessable/unclassifiable. Causality assessment takes into account additional risk factors, such as underlying (maternal) diseases or other drug exposures and the temporal relationship between medication and ADR, including the response to the withdrawal of the drug. It is rarely possible to assess the causal relationship with absolute certainty for any ADR. The assessment was performed independently by three experts (pediatrics, human genetics, gynecology) experienced in clinical pharmacovigilance. The description of the overall clinical situation and co-medication were considered for each ICSR. All three experts have adhered as closely as possible to the aforementioned criteria. Nevertheless, we are aware that expert judgement is to a certain extent subjective and influenced by individual clinical and scientific experience. In case of disagreement, a joint final decision was made.

Due to the lack of comparison cohorts, missing information on potential confounders, the overall limited cohort size and the even smaller sample sizes of particular fetal/neonatal symptoms and drug exposure (intervals), our evaluation was limited to descriptive statistics. Further and advanced (computational) statistical methods usually applied in observational pregnancy outcome studies for quantification and verification of risks appeared inappropriate given the characteristics of the evaluated pharmacovigilance databases.

### 4.5. Participant and Public Involvement

There was no patient or public involvement in setting the research questions, designing the study, or interpretation of the study results.

## Figures and Tables

**Table 1:** NSAID exposure by substance.

NSAID Substance of Study Cohort	Study Cohort *n* = 217 ^1^	NSAID *n* = 195 ^1^	NSAID/PCM *n* = 22 ^1^
	*n* (%)	*n* (%)	*n* (%)
Acemetacin	1 (0.5)	1 (0.5)	0
Acetylsalicylic acid	8 (3.7)	8 (4.1)	0
Carprofen	1 (0.5)	1 (0.5)	0
Celecoxib	1 (0.5)	1 (0.5)	0
Diclofenac	76 (35.0)	64 (32.8)	12 (54.5)
Flurbiprofen	1 (0.5)	1 (0.5)	0
Ibuprofen	22 (10.1)	18 (9.2)	4 (18.2)
Indomethacin	57 (26.3)	56 (28.7)	1 (4.5)
Ketoprofen	8 (3.7)	6 (3.1)	2 (9.1)
Ketorolac	1 (0.5)	1 (0.5)	0
Loxoprofen	1 (0.5)	1 (0.5)	0
Meloxicam	1 (0.5)	1 (0.5)	0
Naproxen	3 (1.4)	3 (1.5)	0
Niflumic acid	5 (2.3)	5 (2.6)	0
Nimesulide	23 (10.6)	22 (11.3)	1 (4.5)
Piroxicam	7 (3.2)	5 (2.6)	2 (9.1)
Sulindac	1 (0.5)	1 (0.5)	0

^1^ The number of NSAID exposures exceeds the number of cases because several study cases were exposed to more than one NSAID. *n*, number of cases; NSAID, non-steroidal anti-inflammatory drug; PCM, paracetamol. Percentages have been rounded to the nearest tenth and therefore do not add up to exactly 100%.

**Table 2:** Prenatal (A) and postnatal (B) study endpoints in relation to 2nd/3rd study medication per trimester.

	Study Cohort (*n* = 219) *n*	NSAID *n*	NSAID/PCM *n*	PCM *n*
**(A)** **Prenatal study endpoints (*n* = 288) ^1^**				
**Stenosis/closure of ductus arteriosus Botalli**	**99 ^3,4^**	**86**	**4**	**9**
2nd trimester	12	10	1	1
3rd trimester	61	56	1	4
2nd and 3rd trimester	15	12	1	2
Not exactly assignable to 2nd and/or 3rd trimester	11	8	1	2
**Fetal cardiac impairment**	**73 ^5^**	**67**	**2**	**4**
2nd trimester	5	5	0	0
3rd trimester	51	48	1	2
2nd and 3rd trimester	11	10	0	1
Not exactly assignable to 2nd and/or 3rd trimester	6	4	1	1
**Fetal death/stillbirth**	**40**	**32**	**6**	**2**
2nd trimester	9	6	1	2
3rd trimester	28	24	4	0
2nd and 3rd trimester	0	0	0	0
Not exactly assignable to 2nd and/or 3rd trimester	3	2	1	0
**Oligohydramnios/anhydramnios**	**76 ^6^**	**63**	**11**	**2**
2nd trimester	24	21	3	0
3rd trimester	30	25	4	1
2nd and 3rd trimester	21	17	4	0
Not exactly assignable to 2nd and/or 3rd trimester	1	0	0	1
**(B)** **Postnatal study endpoints (*n* = 183) ^2^**				
**Neonatal cardiac failure**	**66**	**55**	**4**	**7**
2nd trimester	4	3	0	1
3rd trimester	42	35	3	4
2nd and 3rd trimester	15	14	0	1
Not exactly assignable to 2nd and/or 3rd trimester	5	3	1	1
**Persistent pulmonary hypertension (PPHT)**	**50**	**42**	**4**	**4**
2nd trimester	2	2	0	0
3rd trimester	33	27	3	3
2nd and 3rd trimester	10	10	0	0
Not exactly assignable to 2nd and/or 3rd trimester	5	3	1	1
**Patent ductus arteriosus (PDA) ^7^**	**22**	**17**	**0**	**5**
2nd trimester	3	2	0	1
3rd trimester	16	12	0	4
2nd and 3rd trimester	2	2	0	0
Not exactly assignable to 2nd and/or 3rd trimester	1	1	0	0
**Renal failure**	**26 ^8^**	**21**	**4**	**1**
2nd trimester	2	2	0	0
3rd trimester	13	11	2	0
2nd and 3rd trimester	10	7	2	1
Not exactly assignable to 2nd and/or 3rd trimester	1	1	0	0
**Neonatal death**	**19 ^9^**	**19**	**0**	**0**
2nd trimester	6	6	0	0
3rd trimester	4	4	0	0
2nd and 3rd trimester	8	8	0	0
Not exactly assignable to 2nd and/or 3rd trimester	1	1	0	0

Multiple endpoints in one pregnancy are listed and counted separately. Therefore, the number of individual endpoints exceeds the number of cases in the study cohort. NSAID, non-steroidal anti-inflammatory drug; PCM, paracetamol. ^1^ Prenatal study endpoints were counted per pregnancy. ^2^ Postnatal study endpoints were counted per infant. ^3^ Including eight twin pregnancies. In one, both fetuses were affected, while only one fetus was affected in each of the others. ^4^ In 16 cases, the diagnosis of intrauterine ductus anomalies was only made after birth, such as, for example, through autopsy or neonatal echocardiography. ^5^ In one case, cardiac changes became apparent only postpartum during autopsy. ^6^ Includes pregnancies with NSAID treatment for polyhydramnios. ^7^ In total, 5/22 were born at term, and in 7/22 cases, it was unclear whether the infants were born full-term; furthermore, 10/22 were included although born preterm because they had additional study endpoints. ^8^ Eight twins were included; in three twin pairs, both infants were affected. ^9^ Eight twins were included; in one twin pair, both infants died.

## Data Availability

The authors confirm that all relevant data are included in the article. In the databases used, different levels of access are granted for different stakeholders. Part of the data for the current analyses are publicly accessible. The German Federal Institute for Drugs and Medical Devices (BfArM) as a national competent authority is granted high-level access to the ADR databases EudraVigilance (EMA) and VigiBase (WHO); other institutions and the public are granted a lower level of access. In case of VigiBase, related ICSRs of identified ADR reports were additionally requested from the respective national authorities. VigilanceCentral was the national ADR database of the BfArM until 22 November 2017. All relevant data generated or analyzed during this evaluation are included in this published article and its Supplementary Information Files.

## Supplementary Materials

The following supporting information can be downloaded at https://www.mdpi.com/article/10.3390/ph17121592/s1, Table S1: Criteria for generating the study cohort; Table S2: Exposure to study medication assigned by trimester; Table S3: Indication for study medication; Table S4: Cases exposed to (A) NSAID in the 2nd trimester only and (B) cases exposed to paracetamol in the 3rd trimester reported with defined study endpoints; Table S5. MedDRA Preferred Terms for study endpoint search.
