# Supplementary material for: Do Major Pharmacovigilance Databases Support Evidence of Second Trimester NSAID and Third Trimester Paracetamol Fetotoxicity?
Source: Pharmaceuticals (Basel). 2024 Nov 26;17(12):1592. doi: 10.3390/ph17121592 (PMC11676342; doi:10.3390/ph17121592)
Supplement: Supplementary file 1 [file pharmaceuticals-17-01592-s001.zip › Table S1.pdf]

Table S1. Criteria for generating the study cohort.

Filter 1	Formal exclusion criteria
	• ASA low dose (<300mg/d)/anticoagulation prophylaxis
	• ASA dosage unclear
	• No exposure to study medication during pregnancy course
	• Exposure to study medication in 1st trimester only
	• Unspecified exposure to study medication in pregnancy not assignable with certainty to the 2nd/3rd trimester
	• Only rudimentary information not sufficient for assessment
	• Fetal or neonatal death caused by severe maternal illness or death
	• Duplicate
**Filter 2**	**Content-related exclusion criteria**
	• Drug over dose/suicide attempt
	• Teratogenic or fetotoxic co-medication of significant relevance
	• Other co-medication of significant relevance considered as reason for reporting
	• Relevant maternal infectious diseases
	• Severe or complex maternal illness and/or multiple concomitant diseases
	• Multiple malformations in the fetus/child, (supposed) genetic disease
	• Severe/complex heart defect in the fetus/child
	• PDA in preterm-born infants if no other endpoints were present
	• Duplicate (if it was recognised after filter 1)

All ADR reports identified from VigilanceCentral, EudraVigilance, and VigiBase were individually checked. These criteria were applied in filter 1 and filter 2 to obtain the final study cohort.
