# Supplementary material for: Do Major Pharmacovigilance Databases Support Evidence of Second Trimester NSAID and Third Trimester Paracetamol Fetotoxicity?
Source: Pharmaceuticals (Basel). 2024 Nov 26;17(12):1592. doi: 10.3390/ph17121592 (PMC11676342; doi:10.3390/ph17121592)
Supplement: Supplementary file 1 [file pharmaceuticals-17-01592-s001.zip › Table S2.pdf]

Table S2. Exposure to study medication assigned by trimester

Trimester	Study cohort n=219	NSAID n=183	NSAID/PCM n=20	PCM n=16
	n (%)	n (%)	n (%)	n (%)
**2nd trimester only**	41 (18.7%)	32 (17.5%)	5 (25.0%)	4 (25.0%)
**3rd trimester**	126 (57.5%)	110 (60.1%)	9 (45.0%)	7 (43.75%)
**2nd and 3rd trimester**	37 (16.9%)	30 (16.4%)	4 (20.0%)	3 (18.75%)
**Not further assignable to 2nd and/or 3rd trimester**	15 (6.6%)	11 (6.0%)	2 (10.0%)	2 (12.5%)

n, number of cases; NSAID, non-steroidal anti-inflammatory drugs; PCM, paracetamol.
