# Supplementary material for: Do Major Pharmacovigilance Databases Support Evidence of Second Trimester NSAID and Third Trimester Paracetamol Fetotoxicity?
Source: Pharmaceuticals (Basel). 2024 Nov 26;17(12):1592. doi: 10.3390/ph17121592 (PMC11676342; doi:10.3390/ph17121592)
Supplement: Supplementary file 1 [file pharmaceuticals-17-01592-s001.zip › Table S3.pdf]

Table S3. Indication for study medication.

Treatment indication	Study cohort n=219
	n (%)
Acute pain	82 (37.4)
Chronic and/or inflammatory pain (> 14d)	36 (16.4)
Premature labor/cervical insufficiency	36 (16.4)
Fever/flu	13 (5.9)
Polyhydramnios	11 (5.0)
Combinations of the above	15 (6.8)
Other	2 (0.9)
Indication not available	24 (11.0)

When the study drug was administered for pain, it was determined that an exposure of more than 14 days was considered chronic use.
