# Supplementary material for: Do Major Pharmacovigilance Databases Support Evidence of Second Trimester NSAID and Third Trimester Paracetamol Fetotoxicity?
Source: Pharmaceuticals (Basel). 2024 Nov 26;17(12):1592. doi: 10.3390/ph17121592 (PMC11676342; doi:10.3390/ph17121592)
Supplement: Supplementary file 1 [file pharmaceuticals-17-01592-s001.zip › Table S4.pdf]

Table S4. Cases exposed to (A) NSAID in the 2nd trimester only and (B) cases exposed to paracetamol in the 3rd trimester reported with defined study endpoints

A) Cases exposed to NSAID in the 2nd trimester only

Case	Study medication	Start of medication (GW[1] or trimester)	Exposure time[2] (d)	Dosage (mg/d)/ application	Indication	Study endpoint and diagnosis after start of medication if known (d)	GW at birth and pregnancy outcome	Outcome of study endpoint	Additional information according to case summary	WHO-UMC causality assessment
1	Diclofenac	26/27	6	100 oral	uterine cramps	ductus arteriosus stenosis, fetal cardiac impairment (6)	live-born	recovered	Fetal echocardiography showed a constriction of ductus arteriosus and additional a dilatation of the right ventricle with a mild insufficiency of tricuspid valve. All findings normalized 7 days after medication discontinuation.	Probable/ likely
2*	Diclofenac	26	3	50 rectal	analgesia for amniocentesis	fetus/infant 1: ductus arteriosus stenosis/closure, neonatal death (7)	27, live-born twins	fatal	Analgesia for amniocentesis (in total three times) by recipient twin of chronic TTTS. Neonate died one hour after delivery. Autopsy results: intrauterine severe narrowing or possible closure of ductus arteriosus botalli. No information available on the second twin.	Possible
3	Indomethacin	24/25	2	50 - 100 oral	perioperative tocolysis	narrowing of ductus arteriosus (2)	not known	recovered	Operation due fetal neural tube defect. Event improved within one day of stopping indomethacin.	Possible
4	Indomethacin	24/25	2	50 – 100 oral	perioperative tocolysis	narrowing of ductus arteriosus (2)	not known	recovered	Operation due fetal neural tube defect. Studied endpoint improved within 6 days after stopping indomethacin and after 15 days there was a complete regression.	Possible
5*	Indomethacin	20	49	75 not known	polyhydramnios	fetus 1: ductus arteriosus closure, fetal cardiac impairment, fetal death infant 2: patent ductus arteriosus, neonatal death (49)	27, fetus 1: stillbirth 27, fetus 2: live-born	fatal	Mo/di gemini; Delivery at 27 GW with premature rupture of membranes. Autopsy results fetus 1: Thrombotic occlusion of the ductus arteriosus botalli and biventricular, hypertrophic cardiac enlargement. Placenta with anastomosis at the fetal surface suggestive a TTTS. Infant 2 (died of streptococcal sepsis on day 8 of life): patent ductus arteriosus and hydrops fetalis	Possible (fetus 1); unlikely (fetus 2)
6	Ketoprofen	22	4	not known	rheumatic disorder	narrowing of ductus arteriosus, oligohydramnios, (4)	not known	recovered	All findings normalized after 13 days of medication discontinuation.	Possible

7*	Indomethacin	19	1	200 rectal	perioperative tocolysis	oligohydramnios (56), narrowing of ductus arteriosus (1)	30, live-born	unknown	Medication due to foeticide in gemini pregnancy with pronounced IUGR of one fetus. One day after the operation a ductus arteriosus constriction was detected by ultrasound. Another day later normal blood flow in ductus arteriosus botalli. Several weeks later developing of oligohydramnion.	Unlikely
8*	Indomethacin	19	1	200 rectal	perioperative tocolysis	ductus arteriosus stenosis/closure, fetal cardiac impairment (1)	30, live-born	recovered	Foeticide caused by anomalia of one twin (mo/di) at 19 GW with perioperative indomethacin treatment to prevent labor. One day after operation echocardiographic changes were seen in the remaining twin, which were consistent with ductal occlusion. These changes were no longer detectable just one day later.	Unlikely
9	Diclofenac, Ibuprofen	2nd trimester	not known	unknown (dosage six times per day)	backpain	ductus arteriosus stenosis	37, live-born	unknown	At GW 36 a fetal echocardiography was performed due to fetal hydramnios and showed a normal fetal cardiac structure, but a slight acceleration of blood flow in the ductus arteriosus. Delivery was induced.<br>Concomitant medication: paracetamol; no further information on exposure time.	Conditional/ unclassified
10	Ibuprofen	2nd trimester	not known	not known	not known	ductus arteriosus stenosis/closure, fetal cardiac impairment, neonatal cardiac failure	30, live-born	recovered	Patient was presented to pediatric cardiologist at the 2nd trimester for an asymmetry of the fetal cardiac cavities. An ultrasound scan showed constrictive ductus arteriosus. At 30 weeks of gestation, a further ultrasound revealed a dilated right ventricle, hyperechogenic and hypokinetic. The ductus arteriosus was not detectable and there was a tricuspid leak. The fetus developed a major heart failure and intrauterine premature closure of the ductus arteriosus. Postnatal ultrasound at neonatal intensive care was fine.	Conditional/ unclassified
11	Diclofenac, Ibuprofen, Paracetamol	<27	not known	6 times per day, not known, not known	backpain	ductus arteriosus stenosis (<63)	37, live-born	unknown	Diagnosis at GW 36; after induced labor with oxytocin baby was born with hemangioma in the left hypochondrial (8.5 cm) and the whole body was edematous. Patient was intubated after 5 minutes of live.	Unassessable/ unclassifiable

12	Diclofenac, Acemetacin	2nd trimester	not known	not known oral	chronic polyarthritis	fetal death, oligo-/anhydramnios, fetal cardiac impairment	23, stillbirth	fatal	Additional maternal diagnosis: arterial hypertension, pulmonary tuberculosis, at 22 GW diagnosis of oligohydramnios and intrauterine growth restriction with fetal heart dilatation	Unlikely
13*	Indomethacin	24	21	not known oral	polyhydramnios	fetus 1: fetal death fetus/infant 2: oligo-/anhydramnions, renal failure, neonatal death (21)	27, stillbirth (fetus 1); 27, live-born (fetus 2)	fatal	Additional treatment: multiple amniocentesis. Fetus 1 died in utero shortly before the delivery of twin 2.	Possible
14*	Indomethacin	23, 27	3,3	300, 300 rectal	imminent premature birth	fetus 1: fetal death, fetus 2: oligohydramnios (3)	27, fetus 1: stillbirth 32, fetus 2: live-born	recovered (oligohydr amnios)	Di/di gemini. At 23 GW indomethacin treatment and cerclage due to imminent premature birth. 7 days after stopping medication normalisation of amniotic fluid. At 27 GW rupture of membrans and fetus 1 was born dead. Insertion of a second cerclage and repeated indomethacin therapy. No information on recurrence of oligohydramnios.	Possible
15	Naproxen	23	16	500 oral	thrombophlebitis	fetal death (16)	stillbirth	fatal	After 16 days of treatment intrauterine death was noted. No cause of death was found.	Unlikely
16	Piroxicam	18, 22	11, 20	20 oral	not known	anhydramnios, fetal death (56/70)	28, stillbirth	fatal	Fetus with severe fetal growth retardation and anhydramnios at 26 GW. In addition, placenta was hypotrophic.	Unlikely
17	Ibuprofen, Paracetamol	2nd trimester	not known	200, 1000 oral	rheumatoid arthritis	fetal death	stillbirth	fatal	GW 20: no fetal heartbeat was present on uterine ultrasound. Fetal death occurred; concomitant medication: leflunomide.	Unassessable/ unclassifiable
18	Ibuprofen, Indomethacin	2nd trimester	not known	not known, 50	spondylitis ankylosans	oligohydramnios (35)	not known	recovered	Long-term use of indomethacin and intermittent ibuprofen. Oligohydramnios was diagnosed at GW 27. One month after discontinuation of the medication, findings normalized.	Probable/ likely
19*	Nimesulide	24	25	200 oral	imminent premature birth	Fetus 1 and 2: oligohydramnios (25)	38, live-born twins	recovered	Di/di gemini. Additional treatment: cervical cerclage. In situation with stable cervix and severe oligo with an AFI of 2.2cm and 2.8cm (no history of premature membrane rupture and normal fetal kidneys and fetal Doppler values on US), nimesulide was discontinued, fetuses recovered from oligohydramnios within 14 days. No further events during rest of pregnancy.	Probable/ likely

20	Naproxen	25	5	1500<br>oral	superficial venous thrombosis	oligohydramnios<br>(5)	not known	recovered	After diagnosis of oligohydramnios and umbilical vascular disorder naproxen was discontinued. 2 days later the amniotic fluid index and umbilical blood flow improved.	Probable/<br>likely
21*	Diclofenac	12	75	150<br>not known	rheumatoid arthritis	oligo-/anhydramnios<br>(75)	36, live-born twins	recovered 6 days after diagnosis)	Additional diagnosis: maternal situs transversus,<br>one child: clubfoot	Possible
22	Diclofenac	18	18	150 – 300<br>oral	sciatica pain	oligo-/anhydramnios<br>(18)	36, live-born	recovered	No oligohydramnios was detectable 19 days after discontinuation of diclofenac.	Possible
23	Indomethacin	14	60	not known	adult-onset still's disease	oligohydramnios	32, live-born	recovered	Patient developed a transient and severe oligohydramnios which regressed after stopping indomethacin.	Possible
24	Indomethacin	<25	3 – 15	100 – 200<br>oral	cervical insufficiency	oligohydramnios<br>(3 – 15)	not known	unknown	Patient received indomethacin for cervical insufficiency as part of a study and was monitored for oligohydramnios and changes in ductus arteriosus botalli. She developed borderline oligohydramnios.	Possible
25	Indomethacin	<25	3 – 15	100 – 200<br>oral	cervical insufficiency	oligohydramnios<br>(3 – 15)	not known	unknown	Patient received indomethacin for cervical insufficiency as part of a study and was monitored for oligohydramnios and changes in ductus arteriosus botalli. She developed borderline oligohydramnios.	Possible
26	Indomethacin	<25	3 – 15	100 – 200<br>oral	cervical insufficiency	oligohydramnios<br>(3 – 15)	not known	unknown	Patient received indomethacin for cervical insufficiency as part of a study and was monitored for oligohydramnios and changes in ductus arteriosus botalli. She developed borderline oligohydramnios.	Possible
27	Indomethacin	<25	3 – 15	100 – 200<br>oral	cervical insufficiency	oligohydramnios<br>(3 – 15)	not known	unknown	Patient received indomethacin for cervical insufficiency as part of a study and was monitored for oligohydramnios and changes in ductus arteriosus botalli. She developed borderline oligohydramnios.	Possible
28	Indomethacin	<25	3 – 15	100 – 200<br>oral	cervical insufficiency	oligohydramnios<br>(3 – 15)	not known	unknown	Patient received indomethacin for cervical insufficiency as part of a study and was monitored for oligohydramnios and changes in ductus arteriosus botalli. She developed borderline oligohydramnios.	Possible
29	Indomethacin	>27	not known	not known	premature contractions	anhydramnios<br>(≤ 5)	live-born	recovered	Unexpected pregnancy, arterial myoma embolisation in anamnesis 10 years ago. At 26 GW, patient represented to emergency department with uterine cramps and vaginal bleeding. An ultrasound revealed an intrauterine pregnancy. After	Possible

									beginning with indomethacin treatment uterine cramps didn't stop and some days later due to increasing symptoms an emergency caesarean section was performed.	
30	Naproxen	not known	not known	1100<br>not known	myalgia	oligohydramnios,<br>renal failure,<br>neonatal death	>27, live-born	fatal	At 27 weeks' gestation a massive oligohydramnios was detected. Postpartum the child showed pronounced renal disorder and died on day 30 of life. Autopsy results: renal tubular dysgenesis.	Possible
31	Diclofenac,<br>Paracetamol	22, 19	41, 14	75 -150,<br>2000<br>oral	arthropathic psoriasis	anhydramnios<br>(22)	38, live-born	recovered	Detection of anhydramnios at GW 25. Infilling of amniotic fluids and oligohydramnios afterwards. Amniotic fluids had normalized to the upper norm in 3rd trimester (few days after diclofenac intake was stopped).	Possible
32	Diclofenac,<br>Paracetamol	27, 27	3, not known	100,<br>not known<br>i.m., i.v.	renal colic	oligohydramnios<br>(3)	35, live-born	recovered	48 hours after discontinuation of diclofenac, patient recovered of oligohydramnios; at GW 35 labor was induced because of mild preeclampsia. Concomitant medication: metamizol i.v. (started a few days earlier than therapy with Diclofenac).	Possible
33*	Ketoprofen,<br>Paracetamol	19	18, not known	300,<br>6cp/l	rheumatic disease	fetus 1 and 2:<br>oligohydramnios<br>(18)	not known, twins	recovered	No further information available	Possible
34	Indomethacin	2nd trimester	12	50 – 100<br>oral	cervical insufficiency	oligohydramnios	not known	unknown	No further information available	Conditional/ unclassified
35*	Indomethacin	25	15	75 – 150<br>oral	polyhydramnios	fetus/infant 1:<br>oligo-/anhydramnios,<br>neonatal death<br>fetus 2:<br>oligo-/anhydramnios	Live-born twins	Fatal (infant 1)	Medication due to polyhydramnios at GW 25, both twins born with oligohydramnios.	Conditional/ unclassified (Oligo)
36*	Indomethacin	27	1	200<br>rectal	premature contractions	fetus/infant 1:<br>neonatal death,<br>neonatal cardiac failure,<br>primary pulmonary hypertension<br>(1)	27, live-born twins	fatal	Patient received two indomethacin rectal suppositories (100 mg six hours apart) in case of vaginal bleeding and uterine contractions at 27 weeks of a twin gestation.	Possible
37	Ibuprofen	2nd trimester	not known	not known	not known	patent ductus arteriosus,<br>neonatal cardiac failure,<br>primary pulmonary hypertension	40, live-born	unknown	Additional diagnosis of child: congenital cystic lung, pericardial effusion, incomplete right bundle branch block	Conditional/ unclassified

B) Cases exposed to paracetamol in the 3rd trimester

Case	Study medication	Start of medi-cation (GW[1] or trimester)	Exposure time[2] (d)	Dosage (mg/d)/ application	Indication	Study endpoint and diagnosis after start of medication if known (d)	GW at birth and pregnancy outcome	Outcome of study endpoint	Additional information according to case summary	WHO-UMC causality assessment
38	Paracetamol	30	14	4000 oral	leg pain	ductus arteriosus stenosis, fetal cardiac impairment, patent ductus arteriosus, neonatal cardiac failure, primary pulmonary hypertension (14)	38, live-born	recovered	Restrictive ductus arteriosus and fetal cardiac failure was diagnosed and paracetamol intake was stopped. After one week echocardiographic findings were regressive. A few hours postpartum neonate was admitted to neonatal intensive care unit due to tachypnea and cyanosis. An echocardiography showed hypertrophic right ventricle, patent ductus arteriosus and pulmonary hypertension. Neonate required oxygen for eight days. Pulmonary hypertension resolved, and ductus arteriosus closed spontaneously.	Possible
39	Paracetamol	36	28	not known	back pain	ductus arteriosus stenosis/closure, neonatal cardiac failure, primary pulmonary hypertension 28	live-born	recovered	Paracetamol intake probably at the same time as concomitant medication cyclobenzaprine (reason for ADR report). Postpartum echocardiography at one hour of age showed moderate hypertrophic right ventricle, mild tricuspid regurgitation, elevated right ventricular systolic pressure, two small ventricular septal defects, a small secundum atrial septal defect with right to left shunting and no patent ductus arteriosus. Pulmonary hypertension. The findings normalized within four weeks.	Possible
40	Paracetamol	27	30	not known oral	lower back pain	ductus arteriosus stenosis, fetal cardiac impairment 51	not known	not recovered	In addition, a transposition of the great arteries was diagnosed	Unlikely
41	Paracetamol	27	not known	several days	pain (ileus)	ductus arteriosus stenosis, neonatal cardiac failure	38, live-born	recovered	Hospitalization for abdominal pain. Based on patient's history of 3 laparotomies, ileus was diagnosed. For symptoms pain treatment and tocolysis (not specified in detail) was performed. A fetal ultrasound showed premature constriction of ductus arteriosus. Medication was stopped (exception: tocolysis). Ileus and the constriction of ductus arteriosus improved. Postpartum there was	Unlikely

									only a mild tricuspid regurgitation. Concomitant medication: pentazocine, medication for tocolysis (report from 2017: tocolysis with NSAID not likely).	
42	Paracetamol	38	3	not known	dental abscess	ductus arteriosus closure (3)	38, live-born	recovered	At GW 18 transposition of the great arteries was diagnosed. Further course of pregnancy was uncomplicated until GW 38. After 3 days of drug administration, on echocardiography, fetal characteristics were found closure of the ductus arteriosus and foramen ovale. The modification of hemodynamic situation forced caesarean section. Postpartum surgery was performed on 7th day of life. Child was discharged in good general condition on the 28th day.	Unlikely
43	Paracetamol	2nd trimester unclear, but definitely 3rd trimester exposure	not known	1000<br>not known	backpain	ductus arteriosus stenosis,<br>oligohydramnios,<br>fetal cardiac impairment	not known	recovered	At GW 29 fetal echocardiography for oligohydramnios. The fetus had distinct narrowing of the ductus arteriosus. Paracetamol was stopped and after 7 days; in ultrasound there was no ductal constriction anymore.	Conditional/ unclassified
44	Paracetamol	32	not known	1500 - 3000<br>not known	not known	ductus arteriosus stenosis,<br>fetal cardiac impairment,<br>patent ductus arteriosus,<br>neonatal cardiac failure (few days)	36, live-born	recovered	At GW 32 fetal echocardiography showed pronounced tortuosity of ductus arteriosus botalli and doppler showed increased systolic and diastolic velocities, up to 2 m/sec. At GW 34 fetal echocardiography showed increased systolic and diastolic velocities, up to 2.9 m/sec and enlargement of right hearts chamber without tricuspid insufficiency. At GW 36 cesarean section was performed because of further pathological fetal echocardiography. An echocardiography control, performed in the 3rd day of life, displayed spontaneous closure of the PDA and of the PFO.	Unassessable/ unclassifiable
45	Paracetamol	29	not known	not known	fever	oligohydramnios	unknown	unknown	Secondary diagnosis: malaria infection of the patient	Unlikely
46	Paracetamol	37/38	4	3000<br>not known	pain	patent ductus arteriosus,<br>neonatal cardiac failure,<br>primary pulmonary hypertension<br>(4)	38, live-born	unknown	Patient presented closed ductus arteriosus, severe cardiomyopathy, right ventricular dysfunction and functional stenosis of pulmonary arteries at birth.	Possible
47	Paracetamol	end of 3rd trimester	3	not known<br>oral	kidney stones	patent ductus arteriosus<br>(5)	liveborn	recovered	(concomitant medication: metamizole)	Unlikely

48	Paracetamol	2nd/3rd trimester	not known	≥ 1300 oral	headache, breast pain	renal failure	34, live-born	fatal	Intake of paracetamol throughout pregnancy. During pregnancy, fetus developed polyhydramnios and multiple amniocentesis were indicated. Postpartum neonatal renal failure and the child died in an age of eight weeks	Unlikely

* twin pregnancy; [1] GW, gestational week; [2] Exposure time in days, either continuous or discontinuous medication intake. TTTS: twin-to-twin transfusion syndrome. mo/di: monochorionic diamniotic twin pregnancy; di/di; dichorionic diamniotic twin pregnancy. Case-by-case WHO-UMC causality assessment refers to the listed study medication.
