# Supplementary material for: Do Major Pharmacovigilance Databases Support Evidence of Second Trimester NSAID and Third Trimester Paracetamol Fetotoxicity?
Source: Pharmaceuticals (Basel). 2024 Nov 26;17(12):1592. doi: 10.3390/ph17121592 (PMC11676342; doi:10.3390/ph17121592)
Supplement: Supplementary file 1 [file pharmaceuticals-17-01592-s001.zip › Table S5.pdf]

Table S5. MedDRA Preferred Terms for study endpoint search

Study endpoints	PT-codes
**Prenatal endpoints**	
Abortion late	PT 10052847
Foetal Death	PT 10055690
Stillbirth	PT 10042062
Amniotic fluid index abnormal	PT 10067079
Amniotic fluid index decreased	PT 10075866
Amniotic fluid volume decreased	PT 10063356
Oligohydramnios	PT 10030289
Foetal renal imaging abnormal	PT 10077581
Foetal renal impairment	PT 10078987
Ductus arteriosus premature closure	PT 10049996
Ductus arteriosus stenosis foetal	PT 10013808
**Postnatal endpoints**	
Death neonatal	PT 10011912
Congenital renal disorder	PT 10066875
Renal failure neonatal	PT 10038447
Renal impairment neonatal	PT 10049776
Circulatory failure neonatal	PT 10009196
Congenital pulmonary artery anomaly	PT 10061074
Congenital pulmonary hypertension	PT 10050701
Hypertension neonatal	PT 10049781
Neonatal cardiac failure	PT 10049780
Patent ductus arteriosus	PT 10034130
Persistent foetal circulation	PT 10034708

PT, preferred term within the MedDRA-terminology.
